# Turning the promise of multipurpose prevention technologies into a market reality: a commentary

**DOI:** 10.3389/frph.2023.1181043

**Published:** 2023-11-02

**Authors:** Anita Dam, Jane Schueller, Kevin J. Peine, Jennifer Mason, Emily Dorward, Ashley Vij

**Affiliations:** ^1^ Office of HIV/AIDS, United States Agency for International Development, Washington, DC, United States; ^2^ Global Health Training, Advisory, and Support Contract (GHTASC), Credence Management Solutions LLC, Washington, DC, United States; ^3^ Office of Population and Reproductive Health, United States Agency for International Development, Washington, DC, United States; ^4^ Global Health Training, Advisory, and Support Contract (GHTASC), Public Health Institute, Washington, DC, United States

**Keywords:** HIV prevention, contraception, multipurpose prevention technologies, MPTs, PrEP (pre-exposure prophylaxis), family planning (FP) PrEP-FP integration, dual prevention pill, informed choice

## Abstract

The promise of multipurpose prevention technologies (MPTs) for the prevention of HIV and unintended pregnancy are on the horizon. While many are still in clinical development, others are closer to becoming a realistic, accessible option for users, like the dual prevention pill (DPP). Researchers, governments, donors, and implementers will have to collaboratively address systemic challenges to successfully introduce and scale-up MPTs. To ensure the rollout of MPTs is successful, the global community should address user and country-specific needs, coordinate with advocates and policymakers, and set a realistic plan for product introduction and scale-up that considers the needs of both family planning (FP) and HIV programs, while laying the groundwork for future new product introduction. To achieve these aims, global and regional stakeholder coordination should emphasize country-led, person-centered decision-making while addressing: (1) procurement and supply chain barriers; (2) the potential burden on health systems; and (3) the impact on current programs.

## 1. Introduction

As of 2021, women and girls represent 54% of the 38.4 million people worldwide currently living with the human immunodeficiency virus (HIV), and in sub-Saharan Africa (SSA), women and girls account for 63% of all new HIV infections (1). More than 160 million women and girls who want to avoid pregnancy were not using contraceptives in 2019, with nearly a third of women with unmet need for contraception living in SSA (2, 3). Use of contraceptive methods can substantially improve maternal and adolescent health by averting unintended pregnancy and maternal mortality, supporting women's and girls' empowerment, and contributing to economic and social development (4, 5). Previous analysis mapping has shown that multipurpose prevention technologies (MPTs), which are products that prevent unintended pregnancy and sexually transmitted infections (STIs), including HIV, will have the greatest impact in SSA (6). The demand for combination contraceptive and HIV prevention products by women in these regions is further supported by end-user acceptability studies in multiple SSA countries (7).

The promise of MPTs for the prevention of HIV and unintended pregnancy is on the horizon. Interventions to address this significant overlap of HIV incidence and unmet need for voluntary family planning (FP) are crucial, especially to reach the United Nations Sustainable Development Goal of good health and well-being and the Joint United Nations Programme on HIV/AIDS goal of ending the AIDS pandemic by 2030 (8). Currently, the only available MPTs are male and female condoms, but these are associated with an array of challenges like acceptability, consistent use, partner negotiation, cost, and access (9–11). While many novel MPTs are in pre-clinical and early clinical development, such as intravaginal rings and microarray patches, others like the dual prevention pill (DPP) are closer to becoming a realistic, available option for users (12). The DPP is a daily oral pill combining a hormonal contraceptive and an antiretroviral pre-exposure prophylaxis (PrEP) for HIV, products each of which individually have stringent regulatory approval and are registered in many countries in SSA (13).

Whereas previous commentaries have focused on challenges and opportunities to align MPT research and development, we seek to outline the complex endeavor of “rolling out” an MPT product within a health system and the key issues that must be addressed to make it as smooth as possible (11, 14, 15). Researchers, governments, donors, and implementers will have to collaboratively address the systemic challenges outlined below to successfully introduce and scale-up MPTs. Alignment of investments, regulatory processes, and programmatic vision across HIV and FP–services that have traditionally been siloed–will be essential to prepare existing programming infrastructure for the roll out of the first novel MPT (16).

While many programs are funded to implement single indication products, like oral PrEP and a wide range of short and long-acting contraception, and numerous lessons have been drawn from this work, the first introduction of a novel MPT will undoubtedly serve as a test case, and impact future advocacy, interest, research, and introduction of this new class of products (17–19).

## 2. Key considerations for moving forward with novel MPT Introduction

To ensure the rollout of MPTs is successful, the global community should address user and country-specific needs, coordinate with advocates and policymakers, and set a realistic plan for product introduction and scale-up that considers the needs of both FP and HIV programs, while laying the groundwork for future new product introduction. To achieve these aims, global and regional stakeholder coordination should emphasize country-led, person-centered decision-making while addressing: (1) procurement and supply chain barriers; (2) the potential burden on health systems; and (3) the impact on current programs (20).

### 2.1. Procurement and supply chain barriers

Procurement and structural supply chain barriers to overcome before introducing novel MPTs include policy change and its subsequent operationalization, integration of supply infrastructure, and consistent commodity funding support. For example, the United States President's Emergency Plan for AIDS Relief (PEPFAR) currently does not procure contraceptives, and donor funding for HIV and FP commodities does not always overlap, which can be due to differences in HIV burden and modern contraceptive prevalence rates (21). Moreover, donors have limited funding for commodities, often prioritize a singular health mandate, and/or support existing vertical HIV or FP programs which each tend to have their own separate financing, supply chains, and service delivery systems. For example, the PEPFAR program, while supportive of integrated supply chain systems, is required to achieve HIV specific outcomes and efficiencies that may not be possible through use of national integrated supply chain systems, so parallel quantification, procurement, storage, transportation, and logistics management systems have been established for HIV commodities in many countries.

The addition of a new product to a supply chain system has far reaching funding and technical assistance implications to program areas such as advocacy, planning and forecasting, guidelines development and training, packing, distribution, and data collection and monitoring, among others. To justify pivoting commodity procurement strategies and systems to include new products and taking on the additional costs and work required to introduce and sustain a new product, there must be significant evidence of the value and utility of the product to help achieve health system objectives and meet desires of clients (22). For an MPT, the justification will need to be twofold, illustrating that MPTS benefit both health intervention areas of HIV prevention and FP programs, in terms of cost-effectiveness and increasing access and acceptability relative to the standard of care. Additionally, programmatic considerations such as determining which department of the Ministry of Health will manage and have responsibility for MPTs will affect decisions regarding the funding, inclusion, and distribution of MPTs through an integrated or vertical supply chain.

Since new products are not immediately available in affordable, generic formulations or locally manufactured, the ability of countries to purchase such (brand name) products may be sharply restricted by national and/or donor procurement budgets. This is a particular challenge to MPT introduction, because generic and/or low cost versions of the individual drugs that comprise the MPTs (e.g., oral contraceptive pills and PrEP) are most likely already being procured, have existing rationale and program placement, and have funds and technical assistance allocated to them in countries that are targeted for MPT introduction, which may reduce interest and urgency to introduce a new product. While there is a push for localization of manufacturing, obtaining locally or regionally produced products may be difficult and/or require significant investment. Donors can work with initiatives like the United Nations-backed Medicines Patent Pool (MPP) to facilitate a pathway to generic manufacturing for low- and middle-income countries through patent pooling and non-exclusive voluntary licensing; however, this can take years from initial product availability to generic availability (23).

Often new product development and introduction does not result in increased funds for commodity procurement, meaning countries and donors must make difficult decisions when integrating new products within already constrained budgets. For example, it may be possible for the DPP to be more cost effective than oral PrEP over time, but it is expected to be more expensive than the oral daily contraceptive pill currently used in SSA countries. Financing for this extra cost will likely need to be carried by HIV prevention commodity budgets (20). These decisions can have a major impact on commodity security and availability across the reproductive health space, from the manufacturer to the client (24, 25).

### 2.2. Burden to health systems

As mentioned above, “single issue” funding streams have created or reinforced separate service delivery channels within health systems for what could more appropriately be holistic sexual and reproductive health care. Integrating these separate streams of care provides a comprehensive approach to the user's evolving prevention needs and ultimately results in greater public health impact. However, integrating an established health system, while potentially cost effective in the long term, first requires political will, followed by coordination of national and sub-national management teams, targeted demand generation for clients, supply chain planning, task shifting among facility- and community-based health workers, and capacity strengthening of managers and staff. In particular for the latter, counseling clients on options for FP and HIV prevention while still maintaining voluntarism and informed choice and limiting provider bias will be critical (13, 22).

When integrating an MPT into existing FP, HIV, or integrated service delivery packages, operational considerations at the facility and community levels will need to be updated such as those related to policies and procedures, training, supervision and management structures, health information systems, records keeping, short- and long-term client follow-up, health education, product promotion, and community engagement. Furthermore, complexities within the supply chain management structure like funding, procurement, delivery systems, storage and supply tracking, information management, and training on quantification and forecasting for the new product, will also have to be addressed. Separate from service delivery and supply chain, there is a need for adequate governance and policy coordination to ensure products with multiple indications, often overseen by different departments, are rolled out with both programs in mind (26).

Implementation planning and operations research can tackle many of the outstanding questions for roll out of a new MPT in health systems. Specifically, it could help to identify how to appropriately manage and monitor the introduction and routine provision of a new product, support its eventual scale-up, and guide sustainability efforts. Such research can also provide data for decision-making around the opportunity costs of choosing an MPT over other products in countries with limited donor support and to inform the added value of a new product for potential users.

### 2.3. Programmatic impact

As MPTs come to market, the initial and long-term programmatic impact needs to be considered; hard trade-offs may need to occur when making decisions between MPTs and existing FP and HIV prevention products. Clients may feel influenced to use one product that has multiple indications due to convenience, even if they would not choose the same indications, durations, and/or product type for separate, singular products. Voluntarism and informed choice are cornerstones of FP programming; thus, counseling for both unintended pregnancy and HIV prevention must continue emphasizing choice even with the availability of products with multiple indications vs. singular indications (27). Clients who choose an MPT will have to be supported and monitored through decisions of continued use, switching, or discontinuation, all adding to the time, cost, and capacity of the service delivery structure.

Moreover, FP and HIV prevention programs, often siloed, should consider critical issues surrounding co-delivery of available FP and HIV prevention products in anticipation of an MPT product becoming available soon. Expanding existing programming and integration efforts in the immediate future can be a cost-effective way to make co-delivery a reality and create successful pathways for MPT introduction and scale up (21). Additionally, understanding of sociocultural issues, values, and preferences and prioritization of client perspectives must continue to be considered when integrating FP and HIV services and introducing the added option or choice of an MPT. Among these considerations are gender norms, social norms, intimate partner violence, partner negotiation, and stigma or discrimination that impede access to and use of FP and/or HIV services, including health provider biases (28).

## 3. Discussion

We have outlined three major challenges that need to be addressed to introduce and scale up the first novel MPT efficiently and effectively. In terms of timeline, the novel MPT closest to becoming a market reality is the DPP, and the next step in the successful introduction of this product will be to bring together the global community such as manufacturers, governments, donors, providers, clients, implementers, advocates, and more to create an equitable, ethical, and sustainable plan for roll out and eventual scale up. The global community should also critically evaluate the tradeoffs of rolling out the DPP vs. continuing to have separate, single indication products depending on country priorities and whether the country-context is a good fit.

### 3.1. Bridging research with implementation

As a new generation of prevention technologies like MPTs comes closer to market reality, steps must be taken to bridge research with implementation to meet the needs of clients and achieve the greatest impact. It will be particularly important to bring together the local and global scientific communities to provide data and support to Ministries of Health and other key decision makers to help inform plans for country level procurement, introduction, and scale up of MPT products. Among key decision makers should be representatives from the populations who can most benefit from integrated products and services. Meaningfully engaging women, girls, and civil society advocates ensures a user-centered approach is responsive to the sexual and reproductive health needs and wants of the intended populations for MPTs (29). For example, the PEPFAR/USAID funded MOSAIC project which focuses on the introduction and access of new HIV prevention technologies in SSA, has a team of paid youth advocates under the age of 30 called the MOSAIC NextGen Squad. With members from the 10 countries MOSAIC works in, the group is meaningfully engaged to hold MOSAIC researchers and programmers accountable for ensuring the project's plans, activities, monitoring and evaluation, and learning efforts respond to young people's diverse needs, preferences, and lived experiences, including those of adolescent girls and young women (30).

Lessons from contraceptive research and programming have shown that availability of a greater method mix increases adoption and continuation rates, lessening the unmet need for FP (31). However, it is not easy to simply introduce a new prevention technology into national health systems, as learned from oral PrEP programming (32). Increasingly, implementation science has been identified as an integral approach for new product introduction because it offers frameworks, tools, and methodologies that support the systematic, holistic roll out of a new product using a socio-ecological model (33, 34). Ultimately, insight gained from implementation science studies and related work leads to better informed choice counseling and adherence strategies to respond to clients' evolving needs across their lifetime (35). It further provides national stakeholders and communities with a sustainable pathway to scale up prevention interventions through a continuous, research-to-practice learning process.

### 3.2. Next steps

Current research to inform rollout of the DPP includes formative studies and randomized crossover trials addressing user and provider preferences, acceptability, and adherence in South Africa and Zimbabwe (36). While targeted research-to-roll out efforts are underway to make the DPP the first MPT market reality, there is a need to expand on product-neutral MPT programming that includes coordinated global procurement and integrated approaches to introduction across health disciplines; the following outlined activities should start sooner than later to mitigate any delays in terms of transition from product R&D to introduction. A joint implementation science agenda will be imperative to allow for research results to be shared across countries and regions and to facilitate expedited application of learnings; this agenda should be set and coordinated by a global, normative body like the World Health Organization which has strong relationships with national governments. Governments, donors, implementers, users, and civil society organizations should work hand-in-hand to address procurement and financing barriers now in the early stages of planning, understanding the potential demand and market size for an MPT and changes to the contraceptive prevalence rate and HIV incidence. Investments in MPTs should be integrated and person-centered so the value of a multipurpose product can be realized for users and health systems; thus far, funding opportunities for MPTs has been limited and there is a need for innovative funding and financing strategies to leverage current funding opportunities. Through global coordinated action, successful implementation science, and realistic planning, integrated programs with a wide-range of products, including MPTs, will respond to the needs of women and girls and empower decision-making over their sexual and reproductive health.

## Data Availability

The original contributions presented in the study are included in the article/Supplementary Material, further inquiries can be directed to the corresponding author.
